# Supplementary material for: A Comprehensive Assessment of Ultraviolet-Radiation-Induced Mutations in Flammulina filiformis Using Whole-Genome Resequencing
Source: J Fungi (Basel). 2024 Mar 20;10(3):228. doi: 10.3390/jof10030228 (PMC10971301; doi:10.3390/jof10030228)
Supplement: Supplementary file 1 [file jof-10-00228-s001.zip › Supplementary Material S8/KEGG annotation/out/64381550635650.os/KO/out_map/map04390.html]

KEGG PATHWAY: Hippo signaling pathway - Reference pathway


|  |  |
| --- | --- |
| **Hippo signaling pathway - Reference pathway** |  |

[
Pathway menu
| Organism menu
| Pathway entry
| Show description
| User data mapping
]

Hippo signaling is an evolutionarily conserved signaling pathway that controls organ size from flies to humans. In humans and mice, the pathway consists of the MST1 and MST2 kinases, their cofactor Salvador and LATS1 and LATS2. In response to high cell densities, activated LATS1/2 phosphorylates the transcriptional coactivators YAP and TAZ, promoting its cytoplasmic localization, leading to cell apoptosis and restricting organ size overgrowth. When the Hippo pathway is inactivated at low cell density, YAP/TAZ translocates into the nucleus to bind to the transcription enhancer factor (TEAD/TEF) family of transcriptional factors to promote cell growth and proliferation. YAP/TAZ also interacts with other transcriptional factors or signaling molecules, by which Hippo pathway-mediated processes are interconnected with those of other key signaling cascades, such as those mediated by TGF-beta and Wnt growth factors.

|  |  |
| --- | --- |
| Reference pathway | 100% |
